# Automated semantic annotation of rare disease cases: a case study

**DOI:** 10.1093/database/bau045

**Published:** 2014-06-04

**Authors:** Maria Taboada, Hadriana Rodríguez, Diego Martínez, María Pardo, María Jesús Sobrido

**Affiliations:** ^1^Department of Electronics & Computer Science, ^2^Department of Applied Physics, Campus Vida, University of Santiago de Compostela, ^3^Department of Neurology, University Hospital Clinico of Santiago de Compostela and ^4^Fundación Pública Galega de Medicina Xenómica-Instituto de Investigación Sanitaria de Santiago (IDIS) and Centro de Investigación Biomédica en red de Enfermedades Raras (CIBERER), Santiago de Compostela, Spain

## Abstract

**Motivation:** As the number of clinical reports in the peer-reviewed medical literature keeps growing, there is an increasing need for online search tools to find and analyze publications on patients with similar clinical characteristics. This problem is especially critical and challenging for rare diseases, where publications of large series are scarce. Through an applied example, we illustrate how to automatically identify new relevant cases and semantically annotate the relevant literature about patient case reports to capture the phenotype of a rare disease named cerebrotendinous xanthomatosis.

**Results:** Our results confirm that it is possible to automatically identify new relevant case reports with a high precision and to annotate them with a satisfactory quality (74% F-measure). Automated annotation with an emphasis to entirely describe all phenotypic abnormalities found in a disease may facilitate curation efforts by supplying phenotype retrieval and assessment of their frequency.

**Availability and Supplementary information:** http://www.usc.es/keam/Phenotype Annotation/.

**Database URL:** http://www.usc.es/keam/PhenotypeAnnotation/

## Introduction

Misdiagnosis and non-diagnosis are major obstacles hampering appropriate treatments that could improve quality of life for numerous rare disease patients. Early recognition of this kind of diseases is often vital for timely interventions that can slow disease progression, mitigate its effects and monitor or prevent the known associated complications. When available, the manual revision of practice-based evidence coming from published case reports is the traditional way used by doctors confronted with diagnostic and treatment decisions for their difficult patients. However, the exponentially growing number of peer-reviewed patient cases published in journals, often as case reports, makes it hard to find and contrast information on patients with similar disorders. On the other hand, the availability of high-throughput genetic sequencing technologies is resulting in a wealth of papers on new mutations and syndromes, whose interrelationship will need thorough study in the years to come. Research challenges in the rare disease area include identification of uncommon patients for clinical trials, recognition of the clinical spectrum of a given disorder and understanding genetic and environmental factors that influence disease manifestations (phenotype). Therefore, automated online tools to help find and compare independent case reports scattered throughout the medical literature are much needed.

At world level, over the past few decades, clinical domains have built up extensive experience and knowledge, some of which has been uploaded into one of the leading repositories for scientific literature, PubMed. Unfortunately, the description of this expertise occurs as natural language text, hindering automated searching, analysis and integration of patient data. A great challenge in the use of PubMed information is in the automated retrieval of abstracts or papers relevant to the query. To alleviate this problem, PubMed indexes articles using the standard terminology Medical Subject Headings (MeSH) (1), thereby facilitating their search on specific topics. In addition, PubMed provides filters to limit the search by selecting different criteria, such as type of article and publication dates. In particular, we may limit the search to ‘case reports’ filtering by this type of article. Alternatively, we may use a specialized Web site, which brings all these case reports together, such as ‘CasesDatabase’, http://www.casesdatabase.com. However, not all publications containing significant clinical descriptions have been categorized as ‘case reports’, and thus they cannot be found following this procedure. This is a major drawback in rare disease, where the number of published cases is limited, and therefore it is vital to recover available data from as many cases as possible.

Once reports on patient cases have been recovered, it is fundamental to have good tools to navigate them and ask pertinent questions in efficient nonmanual ways. The search engine used by PubMed presents the results arranged by descending order of PubMed identification number, which is a tedious and user-incomprehensible mode of receiving information. Some recent approaches mitigate this problem by organizing the retrieved information with the use of ontologies, which are domain knowledge descriptions in a computer-processable format. This approach consists of first annotating the key words contained in a document and then scanning the whole document collection to identify which other documents cover some of the same key words. Documents having many words in common are semantically close, whereas those with few words in common are considered semantically distant.

GoPubMed (2) is a successful search engine for biomedical texts, based on the background knowledge of the Gene Ontology (GO) (3) and MeSH. It applies text mining to recognize ontological concepts in the text and to use the MeSH terms provided by PubMed for each abstract. GoPubMed hierarchically organizes the retrieved abstracts taking the GO and MeSH taxonomies into account, thus enhancing the presentation to the user. The SEGOPubmed proposal (4) uses a semantic similarity measure to match the query and the abstracts, instead of term matching. This approach incorporates semantics in the search; however, it requires a corpus of well-referenced key words. GOtoPUB (5) enriches PubMed queries with the descendants of the GO term of interest, thus retrieving abstracts ignored by GoPubMed.

Above all, the quality of the search results largely depends on annotation quality. PubMed curators annotate abstracts manually, ensuring high quality. However, owing to the high costs involved, an informatics challenge is to provide optimal service through automatic annotation with ontologies (6). To ensure a high quality of automated annotation, several main factors must be taken into account, including the accuracy and performance of the software to mine text and the availability of detailed and accurate ontologies to index the data sources. As an example, ‘cerebrotendinous xanthomatosis (CTX)’ is a rare lipid-storage disease causing a variable spectrum of neurologic and other organ dysfunction. A clinician searching for patient cases with ‘CTX’ and ‘intellectual disability’ in PubMed or GoPubMed (at the date of writing this article) would retrieve 27 abstracts missing at least four relevant papers (PubMed Identifiers (PMID): PMID 1124985, PMID 2072121, PMID 6883710, PMID 10768627), where ‘intellectual disability’ is described by synonyms like ‘low intelligence’ or ‘mental deficiency’, which are not covered by MeSH. When searching for patients with ‘CTX’ and ‘epilepsy’, PubMed would return 14 papers, omitting at least several abstracts (including PMID 2265509 and PMID 20329433). The first of these two papers describes two siblings with CTX and ‘febrile convulsions’, and the second presents a case with ‘recurrent generalized tonic–clonic seizures’. Although GoPubMed is able to retrieve these abstracts thanks to the automated annotation, it also retrieves abstracts that do not describe patients with ‘epilepsy’, like PMID 17623518. The reason is that this engine recognizes the string ‘seizures’ (‘epilepsy’ is a synonym of ‘seizures’) from the introduction to disease characteristics at the beginning of the abstract, and it then wrongly annotates the abstract with this label. Therefore, identifying the relevant document snippets for annotating is a needed challenge to improve the quality of automated annotations. Uncurated automated annotations are usually undervalued because of being least accurate; consequently, sometimes users prefer to remove them from their analysis (7–9). Additionally, not only automated annotations are critical but also manual curated annotations, which are often used to propagate predictions in other domains, such as the Biology. Hence, it is critical to provide accurate consistent manual annotations. Recently, some researchers (10, 11) have published a set of guidelines to assist in the annotation of gene products, with the aim of enriching both the quality and quantity of GO annotations.

On the other hand, although MeSH and GO are two of the most used ontologies/thesaurus to date, available ontology repositories like the National Center of Biomedical Ontology (NCBO) Bioportal (12) or the Open Biological and Biomedical Ontologies (OBO) (13) as well as free ontology-based technology are enabling users to combine and adapt all these resources to create the most suitable annotations in specific domains. For example, using the Human Phenotype Ontology (HPO) (14) would be more appropriate than MeSH to annotate human phenotypes for neurogenetic diseases, like CTX. This would present the retrieved information in a more concise and user-understandable form. In the above example of search for patients reported with ‘CTX’ and ‘epilepsy’, by annotating with the HPO, the abstracts would be better organized under ‘generalized seizures’ or under more specific seizure types like ‘symptomatic’, ‘febrile’ or ‘generalized tonic–clonic seizures’.

We hypothesize that (i) a linguistic pattern-based approach is able to retrieve case reports that have not been tagged as such, which can leverage the automated processing of case reports and their relevant snippets of information and (ii) the quality of annotation can be improved using the most suitable available software tools and knowledge resources to the specific domain. The original contribution of this article is 3-fold: we (i) present an automated method for facilitating the search and extraction of relevant snippets of case reports from PubMed, (ii) present how software pieces can be fit into an ontology-based annotator for a specific domain with a reasonable quality and (iii) propose how to evaluate the effectiveness of the method when there is no gold standard available for comparing the results against.

This article is organized as follows. First, we propose a new method to identify case reports concerning rare diseases and to subsequently annotate them for their phenotypic abnormalities in ‘Methods’. For a better understanding of the method, we used CTX as the rare disorder of interest. ‘Results’ describes in detail the results of our method when this was applied to a data set of 515 CTX abstracts selected from PubMed. In ‘Discussion’, we discuss some implications of our work, taking account of other approaches and prospects of future work. Finally, in ‘Conclusions’, we present the conclusions of the work.

## Methods

### Semantic indexing of case reports of a particular disease

The aim of this process is to semantically annotate and index the relevant literature about patient cases describing the phenotypes of a rare disease, in our example, CTX. It does not simply involve recognizing every possible HPO term in all papers on CTX in PubMed, but rather to retrieve and annotate only the papers regarding case reports. Hence, there are a number of challenges faced in this experience: (i) retrieving all patient cases cited in the literature, (ii) extracting the relevant snippets and (iii) annotating these with a quality comparable with human annotation.

**Figure 1.**
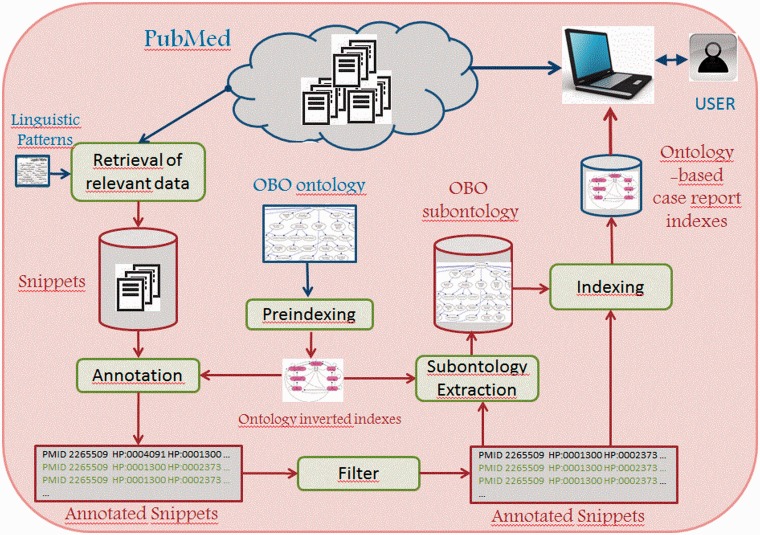
Semantic annotation and indexing of case reports from PubMed.

Using the available abstracts of PubMed (Figure 1), our method extracts the phenotype-relevant snippets of the case reports on CTX. Next, it annotates them with the HPO ontology. After filtering some incorrect annotations, a minimal CTX-specific subontology is induced from the complete set of annotations, and a case report index is generated.

### Retrieval of relevant snippets of patient cases

As it is usual in technical texts, the literature about case reports uses a limited set of linguistic structures to organize and strengthen discourse, thereby reducing ambiguity in communication. Under the preliminary assumption that the description of patient in case reports usually has a modular configuration, easily identifiable, we designed a simple methodology to find these structures we call linguistic patterns. First, a reduced set of abstracts was randomly selected from the complete set of case reports about CTX. Then, these abstracts were analyzed to identify structures used to introduce a case. Examples of these structures are the following:

**In the present *[study|report]* we *[reviewed|examined|…]***

      ***[<an age>] [patients|male|…].***

**A case *[study|report]* on* a [<an age>] [patient|male|…] is***

       ***[described|presented|…]***

**Figure 2.**
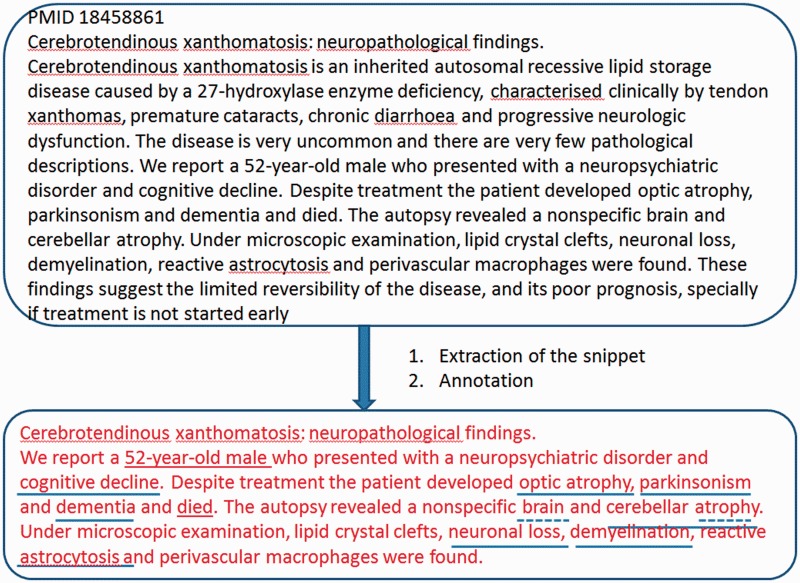
Example of extraction of a snippet of information from an abstract and its subsequent annotation.

Hence, a tentative list of seed structures was drawn up and then used to search for sample sentences in another different small subset of CTX case reports. The result of this second search allowed us to adjust the seed structures to get the patterns to be used. Only the seed structures with a high success rate and low noise were selected as valid patterns. Next, we implemented this set of designed patterns as a separate script to extract the relevant snippets from the abstracts. For this purpose, the algorithm searched for the first occurrence of any pattern within the abstract, analyzing the sentences of the abstract sequentially. Figure 2 shows an example of extracting a relevant snippet from a PubMed abstract.

### Annotation of relevant snippets

Direct HPO annotations on snippets were created using our own annotator called OBO annotator and the annotator provided by the NCBO Bioportal (15).

#### The OBO annotator

It was specifically implemented to annotate biomedical literature with HPO phenotypic abnormalities. Still, it can be applied to recognize terms from any OBO ontology, as it is mainly a name entity recognizer, which matches input text against terms from an OBO ontology.

To optimize the performance of the annotator, the execution time and the required memory space, inverted indexes are used. These are index data structures that map from content (i.e. sequence of words in the text to be annotated) to the corresponding concept in the ontology, thus enabling the annotator to find OBO concepts quickly. Two types of inverted indexes are prebuilt from the OBO ontology: lexical and contextual. Lexical indexes come from processing the preferred terms and synonyms of each concept in several steps: transforming terms into lower case, splitting into tokens, removing common words and punctuation marks, replacing tokens for the corresponding roots (by removing suffixes), generating term variation as permutations of the lexemes and filtering incorrect term variations. Contextual indexes are built by computing the transitive closure over all hierarchical structure of the OBO ontology. They facilitate the retrieval of the all ancestors and descendants of each concept.

The OBO annotator matches sequences of up to a given number of words in the text to the lexical indexes. Before matching, these words are preprocessed as the terms were in the lexical indexes. Next, a sliding window on the preprocessed text extracts sequences of words, which are matched to lexical indexes. In the case of no exact match, the sequence is cut up into smaller subsequences, which are matched. For example, in Figure 2, the sequence ‘brain [and] cerebellar atrophy’ cannot be matched to any term in the dictionary. Hence, the algorithm cut it into smaller subsequences such as ‘brain atrophy’ or ‘cerebellar atrophy’, both of which are matched to terms in the dictionary. Finally, the algorithm filters overlapping annotations, choosing the longest ones. Hence, the OBO annotator never generates the annotation ‘seizures’ when it also recognizes ‘atonic seizures’ in the same text.

#### The NCBO annotator

It is available over the Web, and it can map free text to terms from any of the 270 biomedical ontologies stored in the NCBO’s Bioportal, allowing to expand the annotations with more general terms to the identified terms into text. The core is Mgrep, a concept mapping engine based on an efficient string-matching algorithm.

### Filtering of incorrect annotations

With the aim of verifying the feasibility of the OBO annotator before implementing the complete system, a proof of concept was carried out with an initial version. The evaluation of this proof was conducted by the two neurologists from the working team (M. Pardo and M.J. Sobrido). During the evaluation, they identified a recurrent error: the word ‘xanthomatosis’ was always annotated with the HPO term HP:0000991. However, the string ‘cerebrotendinous xanthomatosis’ should not be annotated with the phenotype HP:0000991. Hence, we decided to add a new step to our method to remove these recurrent incorrect annotations. This step was developed as a filter, which removes the annotation HP:0000991 when it is linked to this string. This particular filter is specific to the CTX domain. In addition, an explicit filter was programmed for the NCBO annotator, as this annotator generates overlapping annotations. On the contrary, this type of filter is not dependent on the clinical domain.

**Figure 3.**
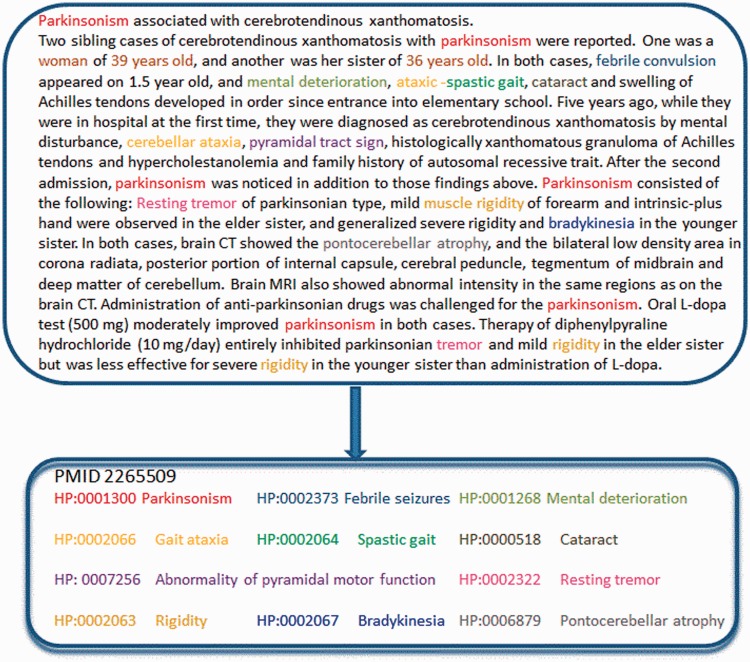
Example of annotation generated by the OBO annotator, using the HPO ontology.

Finally, the algorithm removes repeated annotations, and it subtracts the most general ones, on the basis of the contextual indexes. The upper part of Figure 3 displays the direct annotations recognized by the OBO annotator on the abstract PMID 2265509, which are reduced to the minimal set of phenotypes showed in the lower part. As an example, the direct annotations ‘cerebellar ataxia’ or ‘tremor’ (at the top) were removed from the relevant set of annotations (at the bottom), as other more specific annotations already exist (‘gait ataxia’ and ‘resting tremor’, respectively).

### Extracting the minimal subontology

To remove unessential fragments of the ontology, which are not relevant to the set of case reports, we looked for the minimal subontology including all the terms extracted from the snippets. Thus, we built the minimal subontology by searching all middle concepts that were included in all paths of the ontology, from the extracted terms to the root.

### The evaluation process

Evaluating the quality or performance of the method is really difficult because of the lack of a gold standard reference. Furthermore, the use of manual reviewers is error prone and labor intensive. In view of this situation, we suggest an evaluation structure heading for presenting (i) the advantages introduced by our proposal in one of the potential applicative scenarios (e.g. phenotype-based annotation of a rare disease) and (ii) the benefits of using the automated annotations in revising current releases of curated annotations as well as the annotation ontology.

#### Evaluation against papers tagged as ‘case reports’

As a means of attenuating the workload linked to the review procedure, without lowering evaluation quality, both the complete set of CTX papers available in PubMed and the reduced set of CTX papers tagged as ‘case reports’ were used. We measured the percentage of common and unshared papers between the results obtained by our automated method and the set of papers tagged as ‘case reports’ (which we will call manual method). The two neurologists from the working team manually checked the title and abstracts of the CTX papers tagged as ‘case reports’, and they classified them as correct/relevant or incorrect (when the abstract did not describe CTX patient cases). They manually checked the papers proposed by our method that were not tagged as ‘case reports’, and they classified them as relevant when describing a clinical case of CTX.

In this context, we define precision as the fraction between the number of correct papers and the total number of papers proposed by each method (manual and automated); and recall as the fraction between the number of correct papers proposed by each method and the total number of relevant papers. With the aim of comparing systems, a standard way to combine these two measures in information retrieval is the F-measure, which is a balanced harmonic mean of the precision and recall.

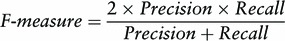



#### Evaluation of annotation relevance

We performed the evaluation in two ways. First, we manually evaluated the precision and recall of the automated annotation, revising 50 abstracts randomly chosen from the snippets extracted by our method. Second, we automatically compare automated annotations to the set of curated phenotype annotations about CTX provided by The HPO consortium (14).

## Results

Our data set involved 515 abstracts selected from PubMed corresponding to papers with the key word ‘Cerebrotendinous Xanthomatosis’ in the title/abstract (at the end of October 2013) and a subset of 223 abstracts limited to case reports. Only the title and the snippets of each abstract directly associated to the patient case were taken into account for annotation. By restricting to the specific ontology HPO, we set out the annotation to the domain of human phenotypes in neurogenetic diseases.

### Selection of case reports and extraction of snippets

From a set of 50 abstracts randomly selected from the 223 abstracts limited to case reports, a tentative list of seed structures was used to search for sample sentences in another different subset of 50 abstracts. A set of five linguistic patterns (see Supplementary Information) was designed from the seed structures with the highest success rate and lowest noise. This set of designed patterns was used to extract the relevant snippets from the complete data set of 515 abstracts.

Table 1 shows the results of the evaluation when the manual and automated method were applied. In Table 1, we can see that our automated approach achieved 99% precision as compared with 97% for manual method. The values of recall were significantly lower: 65 against 81% of the manual method.

**Table 1.** Evaluation results of the performance of the identification of case reports

Evaluation measure	Manual method	Automated method
Number of selected papers	223	174
Precision (%)	97	99
Recall (%)	81	65
F-measure (%)	88	78

### Annotation Relevance: Quality of annotations

Three different tests were conducted using (i) the OBO annotator with HPO, (ii) the NCBO annotator with HPO and (iii) the service provided by GoPubMed, which is based on GO and MeSH. The first two tests were run on all 230 snippets of abstracts [174 extracted by our method plus 56 snippets tagged as ‘case reports’ with no abstract (they only had text in the title section)]. These tests allowed us to compare the OBO and NCBO annotators, as they were used with the same ontology HPO. Because we could not ensure that the two annotators were using the same version of HPO, each time a disparity between the two annotators was identified, we manually verified if the concept annotated by one annotator could be annotated by the other using the same synonym. Thus, we tested that the two annotators were using the same version of HPO, at least as for the CTX domain.

Of the 230 snippets of abstracts (Table 2), at least one concept was recognized in the abstract or title of 145 (63%) by the OBO annotator and of 126 (55%) by the NCBO annotator. On average, 3.3 concepts were annotated per abstract with a standard deviation of 2.56 by the OBO annotator, whereas 2.9 concepts with a standard deviation of 2.05 by the NCBO annotator. The maximum number of concepts discovered per abstract by the OBO annotator was 11, whereas by the NCBO annotator, it was 8. Finally, the total number of annotations recognized by each annotator can be viewed in the last row: 456 (the OBO annotator) against 344 (the NCBO annotator). In total, there were 326 (71%) overlaps, 18 (4%) differences and 112 (25%) extra OBO annotations, when compared with NCBO annotations.

**Table 2.** Annotation results for the OBO annotator and the NCBO annotator

Annotation result	OBO annotator	NCBO annotator
Number of annotated abstracts	145	126
Percentage of annotated abstracts (%)	63	55
Average number of concepts per abstract	3.3	2.9
Standard deviation	2.56	2.05
Maximum number of concepts per abstract	11	8
Total number of annotations	456	344

The reasons for 25% extra OBO annotations and 4% more specific annotations were analyzed. Several factors came into play: replacing tokens for the corresponding roots (13.4%), cutting sequences of text into subsequences (10.2%), using ‘related’ HPO synonyms in addition to exact synonyms (2.9%) and removing common words and punctuation marks (2.6%). We also studied the false-positive rate linked to the characteristics incorporated in the OBO annotator. Only 17% extra and more specific OBO annotations were wrong owing to several causes: incorrect synonym included into HPO (7.5%), use of roots instead of tokens (4.3%), use of term variation (3.2%) and cutting sequences of text into subsequences (2.1%).

Additionally, not all snippets were annotated. This is due, in part, to the fact that some of them do not have abstracts available (that is, only the title is available), and others do not describe the phenotypes of the patient in the abstract. However, even though both annotators are capable to recognize many systematic and neurologic signs, mainly for the case of the OBO annotator, they also fail to recognize physiology and neurophysiology characteristics, as well as some morphologic and biochemical abnormalities. In general, abnormalities obtained from tests conducted in the laboratory are not reported using a single standard term but using sentences including different aspects such as the finding site, the type or degree of the lesion and the technique used. Thus, an algorithm based on name recognition alone is not enough for annotating the complete set of phenotypic abnormalities.

For the third test, as we did not have access to the annotator used by GoPubMed, we manually annotated 50 randomized abstracts from the results seen on GoPubMed’s Web site. It should be noted that GoPubMed works on both the annotations automatically made throughout the abstracts plus manual annotations made by the curators of PubMed. Nevertheless, we only took the automated annotations into account to ensure the same conditions with the other annotators. Comparing the results with this third test was useful to show the effect of using different ontologies.

In Table 3, we show a comparative of the results, with three evaluation measurements: the average number of concepts per abstract (coverage), precision and recall on 50 randomized abstracts. On average, 3.86 concepts per abstract were recognized by the OBO annotator, 3.14 by the NCBO annotator and 2.54 by the GoPubMed’s annotator. Furthermore, the OBO annotator achieved 94% precision, a slightly lower value than the other annotators (97%). But the main difference between our annotator and the others is in recall and F-measure. Recall for our method is considerably higher: 61 versus 49 (the NCBO annotator) and 41% (the GoPubMed’s annotator); as well as F-measure: 74 versus 65 and 58%, respectively. As we have just remarked above, the two main reasons for extra OBO annotations and so, a higher recall, were replacing tokens for the corresponding roots and using cutting sequences of text into subsequences. Although we do not know exactly how the GoPubMed annotator works, the results show the same trend (roots and subsequences) as when the NCBO annotator was compared with the OBO annotator. Additionally, the coverage and recall of GoPubMed is lower than those of the OBO and NCBO annotators, as it is based on a different terminology, which is not specific for the human phenotype domain.

**Table 3.** Evaluation results of the performance of our method, the NCBO annotator and the GoPubMed service

Measure	OBO annotator	NCBO annotator	GoPubMed
Coverage	3.86	3.14	2.54
Precision (%)	94	97	97
Recall (%)	61	49	41
F-measure (%)	74	65	58

### Annotation Relevance: overlapping with the curated annotations

We compared curated and automated annotations through their induced ontologies. The ontology induced by automated annotations covers 324 HPO concepts, whereas the curated ontology covers 137 concepts. In total, both ontologies share 121 HPO concepts. Both ontologies are available at http://www.usc.es/keam/OBOAnnotator/.

## Discussion

Conventionally, clinical research has focused on diseases concerning to the wider patient population. The current scientific understanding of human biology at the molecular level has welcomed the study of diseases at a more individual level. To develop more targeted treatments, establishing smaller clusters of diseases sharing common characteristics is a challenge. Rare diseases may play an important role as tools to figure out fundamental disease mechanisms (The Foundation for Fundamental Diseases, http://www.findacure.org.uk). Providing computational tools oriented to automatically extract phenotypes from patient clusters sharing common characteristics, can make the study of disease much easier. In particular, semantic indexing facilitates synthesis and filtering information from multiple, large and fast-growing sources. Nevertheless, nowadays semantic annotation is mostly achieved manually. Hence, an informatics challenge is to automate it to manage the huge volume of new information being available every day (6). Our work is focused on the semantic indexing of a particular domain: case reports from the literature. Our results confirm that it is possible to extract relevant snippets of information from abstracts of peer-reviewed patient cases reported in the medical literature. Our techniques generate a semantic index of de-identified patient data, which can be migrated and analyzed with more specific methods if needed.

### Findings and significance of the selection of case reports

**Figure 4.**
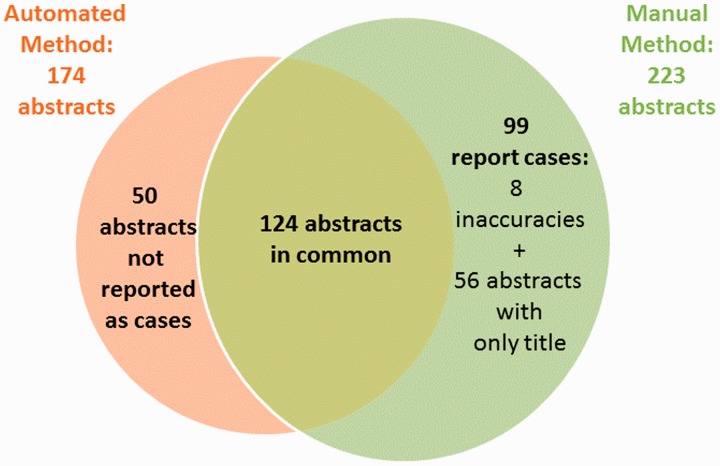
Venn diagram showing where the two methods overlap.

**Figure 5.**
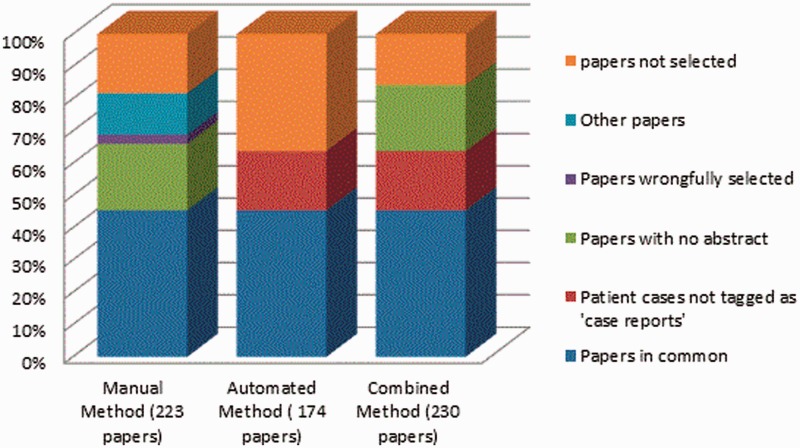
Percentage of papers selected by each method.

With a recall significantly lower than manual method, the performance of our method seemed insufficient. We performed an analysis and identified the following reasons for failure. By applying the automated method to the 223 papers selected by the manual method, only 124 (55%) papers tagged as ‘case reports’ were selected by our method (Figure 4). Additionally, our method identified other 50 papers that had not been tagged as ‘case reports’. Revising the 99 papers selected by the manual method but not by our method, we identified 56 papers with no abstract available (only title available), 8 papers not describing CTX cases and other 35 papers correctly tagged as ‘case reports’. Because our method is based on the processing of the complete abstracts, the 56 papers with no abstract available could not be identified automatically. Hence, we decided to automatically add these ‘case reports’ with no abstract available in PubMed to the results of the automated method, and we called it combined method. In this way, the total number of papers selected was 230 (against 223 of the manual method). Once again, the two neurologists from the working team manually evaluated the results. The combined method achieved 99% precision as compared with 97% for manual method and 87% recall against 81% of the manual method (Table 4). Figure 5 shows the percentage of selected papers by each method.

**Table 4.** Evaluation results of the performance of the identification of case reports for the three methods

Measure	Manual method	Automated method	Combined method
Number of selected papers	223	174	230
Precision (%)	97	99	99
Recall (%)	81	65	87
F-measure (%)	88	78	93

Our results shows that using a set of linguistic patterns based on the regularities observed in the expression of patient information in clinical abstracts, we were able to identify 50 more case reports that had not been tagged as such (Figure 4). This result is crucial in rare disease, where the number of cases is limited, and it is important to recover as much cases as possible. A main advantage of our method is the high precision to automatically identify new relevant cases, with the possibility to use it as a complement of the manual identification of case reports. However, the most significant contribution is the recognition of the snippets relevant to annotation, as this fact cause a direct increasing of annotation accuracy.

Although we have shown in this work that the achieved set of linguistic patterns is a valuable resource for the recognition of the relevant snippets in the domain of CTX, we certainly cannot say that these same patterns will be appropriate in other domains of rare diseases. As a preliminary validation step, we tested these patterns on randomly selected 50 abstracts from Huntington’s disease and on 50 abstracts from Friedreich ataxia. In the first case, 95% precision and 67% recall were achieved, whereas in the second case, 99% precision and 25% recall were achieved. Hence, our set of linguistic patterns cannot be directly used in other rare disease domains, but they could be valid as seed structures.

### Quality of annotation

Unlike most other works about semantic search, we focus on evaluating the annotation process, as it is the core of tools using ontologies for literature exploration. In this context, it should be stressed that there are no definitive studies showing the quality of ontology-based annotation, excepting for the GO (16).

Obviously, our results show that the quality of the search results depends on both the efficacy of extracting relevant snippets of information and the annotation mechanism. The services supplied by the NCBO and GoPubMed offered us a benchmark to evaluate the enhancement of performance of our method. The three annotators used in our study are based on concept recognition. A previous comparative evaluation between MetaMap and Mgrep (17) showed that concept recognizers have clear advantages on addressing speed, flexibility and scalability, when compared with natural language processing (NLP) tools. One of the main contributions of our work is a detailed assessment of the annotation results. These provide valuable performance measures in addition to awareness about the limits of the approaches and how they could be enhanced. Evaluation indicated that annotation quality was satisfactory (74% F-measure when the OBO annotator was used; Table 3). Although the details of how Mgrep works are not completely clear from the publications, and we are not sure if the annotator used by the GoPubMed is exactly the same one followed in (2), the main difference of our annotator is (i) the enriching lexical preprocessing of the OBO ontology terms (offline) and text (online) and (ii) the extraction of sequences and subsequences of words with the sliding window on the preprocessed text. The lexical preprocessing has been implemented by fitting some software pieces already implemented (such as Porter stemming algorithm, generating term variation as permutations of the lexemes) and some available resources (such as stop words or English adjectives). Because this preprocessing does not make use of NLP techniques, features like speed, flexibility and scalability are preserved, while producing an increase at 12 and 20% in recall (Table 3), compared with the NCBO and GoPubMed services, respectively. In total, 74% F-measure may be considered a good result, as the method has been applied without using other techniques. Note that the preprocessing was designed to be applied to any OBO ontology but not to the specific characteristics of the HPO ontology. Preprocessing specific to the characteristic of the HPO ontology would increase recall. For example, substitution of frequent words of HPO terms for synonyms, such as ‘abnormality’ for ‘lesions’, or enriching the lexical indexes with terms from other ontologies, by using the OBO ‘xref’ property, whose primary role is to set mappings between concepts from different ontologies. Preliminary experiments done using the xref property (to the Unified Medical Language System (UMLS) and MeSH) have revealed that it is important to start from curated mappings. The first case (with uncurated UMLS xref) led to numerous errors, whereas in the second case (with curated MeSH xref), the dictionary was enriched with some new synonyms.

One of the awareness in our effort is that phenotype names are hardly longer than four words, and it is computationally manageable to execute a full search for all potential permutations of four or fewer words into the text to be annotated. This is especially true when we try to annotate most of the systematic and neurologic signs, but it mainly fails to recognize physiology and neurophysiology characteristics, as well as some morphologic and biochemical abnormalities. In such cases, it seems more appropriate to use co-occurrence-based techniques after the name recognition has been completed. Additionally, this subsequent step would allow to update the support ontology.

### Comparison with curated annotations

As previously mentioned (see ‘Annotation relevance: overlapping with the curated annotations’), the total coverage of the ontology induced by automated annotations (324 concepts) is higher than the one of the curated ontology (137 concepts). This result was expected as the main attractiveness of automated annotations is just that they scale well to huge amount of data. Table 5 lists the set of HPO concepts induced from annotations in the literature and not present in the curated annotations (although these include some more general concept). In this case, only those phenotypes mentioned at least in four abstracts were involved into the comparative study. Hence, we did not take unusual phenotypes into account. All annotations leading to the concepts in Table 5 were manually revised, and only two cases were erroneous (rows 3 and 10). They correspond to the concepts ‘congenital cataract’ and ‘peripheral demyelination’. The first one has ‘bilateral cataract’ as a synonym in HPO and the second one, ‘demyelination’. As a result, the phenotype ‘bilateral cataract’ is always annotated as ‘congenital’ cataract, and a ‘central demyelination’ is annotated as ‘peripheral demyelination’, which is clearly wrong. The remainder 11 concepts were correct, and they could be considered as candidate phenotypes to be added in newer releases.

**Table 5.** Set of concepts that are more specific in the ontology induced from the literature than in the curated ontology

HPO concept	Correct annotation?	Recommendation
Abnormal emotion/affect behavior	Yes	Yes
Chronic diarrhea	Yes	Yes
Congenital cataract	No	Revise synonyms
Gait disturbance	Yes	Yes
Global developmental delay	Yes	Yes
Juvenile cataract	Yes	Yes
Lower limb spasticity	Yes	Yes
Paraplegia/paraparesis	Yes	Yes
Parkinsonism	Yes	Yes
Peripheral demyelination	No	Revise synonyms
Polyneuropathy	Yes	Yes
Progressive neurologic deterioration	Yes	Yes
Spastic gait	Yes	Yes

Table 6 shows the set of HPO concepts present in the curated annotations and not induced from annotations in the literature. We manually revised the set of abstracts, searching for any term describing these concepts. We could not find nine phenotypes in the abstracts. Maybe if we had analyzed the complete papers, we had annotated these phenotypes, as they are often characterized in the clinical descriptions of CTX (18). From the rest of the concepts, the main reason for the omission is that the concepts have different names in the HPO and PubMed abstracts. In some cases, there are multiples ways to express the concept. For example, ‘abnormality of the dentate nucleus’ can be also described as a ‘lesion’ or ‘hyperintensity of the dentate nucleus’. Once again, replacing recurrent words of HPO terms for synonyms, such as ‘abnormality’ for ‘lesions’ would allow for these terms to be recognized. In other cases, there are more suitable ways to express the concept. For example, a simpler way to express ‘electromyography (EMG): axonal abnormality’ is by ‘axonal abnormality’. Similarly, the HPO term ‘Abnormality of central somatosensory evoked potentials’ is a long series of words to be difficult to appear in texts. As previously mentioned, in cases where abnormalities come from tests lead in the laboratory, an algorithm based on name recognition alone is not enough for annotating the complete set of phenotypic abnormalities. In the future, we plan to use co-occurrence-based techniques to be able to annotate these types of phenotypes.

**Table 6.** Set of concepts included in the curated ontology and not in the one induced from the literature

HPO concept	Is it in the abstracts?	Reason for the omission
Abnormality of central somatosensory evoked potentials	Yes	A long series of words
Abnormality of the dentate nucleus	Yes	Different name
Abnormality of the periventricular white matter	Yes	Different name
Angina pectoris	No	
Cerebral calcification	Yes	Different name
Delusions	No	
Developmental regression	No	Different concept
Electroencephalography with generalized slow activity	Yes	A long series of words
EMG: axonal abnormality	Yes	Different name
Hallucinations	No	
Limitation of joint mobility	No	
Lipomatous tumor	Yes	Different name
Malabsorption	No	
Myocardial infarction	No	
Respiratory insufficiency	No	
Xanthelasma	No	

### Remaining challenges

In our study, we decided on using abstracts instead of full-text papers, as more than half of the latter are not of free public access, and among the available ones, a majority requires to be transformed from PDF format. As a consequence, 9 of the 137 concepts (6.5%) in the curated ontology could not be found in the literature of CTX case reports. This restricted test hints at a large number of CTX-relevant phenotypes can be recognized based just on the abstracts.

### Related work on semantic annotation

Semantic annotation attaches information (names, attributes, comments, descriptions, etc.) to a document or to a selected part in a text, thereby providing metadata about an existing piece of data. Several methods have been proposed with the aim of either partially or completely automating semantic annotation. The procedure is usually viewed as classical named entity recognition (NER) followed by traditional annotation (19). In addition, as ontologies are available to different communities, the flat list format of the named entities sets regularly used in NER has been replaced by the hierarchies provided by one or multiple ontologies. In many cases, NER is performed by NLP, and the GATE framework (the General Architecture for Text Engineering; 20) is the most widely used resource. GATE provides facilities (tokenizers, part-of-speech taggers, pattern-matching grammars, etc.) to develop and distribute software modules processing natural language. For example, the KIM (Knowledge and Information Management) platform (19) offers a semantic annotator based on GATE and KIMO, a formal upper-level ontology. Recently, one semantic platform for cloud service annotation (21) and another in the e-learning domain (22) have been developed using GATE to recognize named entities of multiple ontologies. The first platform applies statistical approaches based on the syntactic structures of the text to disambiguate the entities recognized by GATE, whereas the second one expands the recognized annotations in the text with a graph, which facilitates the access and navigation of the learning contents.

Although NLP-based annotation tools can achieve good quality results, they need huge memory and computational resources. To bridge the gap, there are other alternatives where linguistic analysis techniques are replaced by other procedures. SemTag (23) annotates large-scale Web pages with terms from an upper-level ontology called TAP (Towards a web of data). Texts are tokenized and then processed to find all instances of the ontology. Each candidate annotation is saved with 10 words to either side (named context). Simultaneously, a representative sample of the Web pages is then scanned to determine the contexts at each concept in the ontology. A vector-space model is used to disambiguate candidate annotations by comparing their contexts with the concept contexts in the ontology. This approach presents high accuracy because it dynamically builds the contexts in the ontology using the same corpus to be annotated. However, it also needs huge memory and computational resources.

Other approaches depend mainly on regular expressions (24) or context-free grammars (25) to recognize named entities. In this sense, they are light-weight, but they require building the grammar for each application. One more alternative found in the biomedical domain involves recognizing named entities by exploiting the rich set of the concept synonyms in biomedical ontologies. This is the approach proposed by the NCBO annotator (12), which applies an efficient string-matching algorithm (Mgrep) to find these synonyms inside the text, supplying higher speed, precision, flexibility and scalability than NLP-based annotators (17). Our approach is based on this idea. It is a light-weight tool, with a little lower precision than the NCBO annotator. However, the recall has been significantly improved because of the enriching lexical preprocessing of both the ontology synonyms and text strings, and by extracting subsequences of words on the preprocessed text. Although the OBO annotator has only been tested in the CTX domain, the current implementation can be deployed in other neurologic disease domains without having to make major changes to the source code, as it is based on the HPO ontology. Filters specific to the domain are the only expected changes. Additionally, the OBO annotator is highly customizable to be used with other different OBO ontology. Preprocessing the new ontology, using the same code for HPO, is the only prerequisite required to be ready to use with the annotator.

### Future applications

Our approach to annotate phenotypes was never conceived as a solution to automatic curation of phenotype knowledge, as curators usually handle additional knowledge and information to characterize the influence of a given phenotype on a disease. Still, we claim that approaches like ours facilitate curation efforts by supplying phenotype retrieval and assessment of their frequency. The annotation resourcefulness described here provides a distinct improvement for analysis of phenotype sets. Annotating with an emphasis to entirely describe all phenotypes found in a disease ensures that phenotypes that may have been ignored in other annotation contexts may be curated as relevant. An example of advantage of the automated annotation strength at an individual phenotype level is for the phenotypes ‘gait disturbance’ or ‘parkinsonism’, which have been often associated with CTX in the literature of case reports. As seen earlier, our approach identifies almost all phenotypes mentioned in the literature of interest.

## Conclusions

The proposal has been applied to automatically annotate phenotypes from the set of abstracts stored in PubMed about CTX. Still, we think the proposed methodology to design the OBO ontology-based annotator and evaluate the results is sufficiently generic to be applied for annotating the literature related to any human phenotypic abnormalities of neurogenetic diseases, as the OBO annotator has been restricted to the specific ontology HPO. Significantly, we extensively evaluated the method and showed that when annotators are set properly with the most suitable ontologies to the domain, high-quality annotations with few false-positive findings are reached.

## Supplementary data

Supplementary data are available at *Database* Online.
